# Overcoming Reproductive Compromise Under Heat Stress in Wheat: Physiological and Genetic Regulation, and Breeding Strategy

**DOI:** 10.3389/fpls.2022.881813

**Published:** 2022-05-13

**Authors:** Min Li, Jiming Feng, Han Zhou, Ullah Najeeb, Jincai Li, Youhong Song, Yulei Zhu

**Affiliations:** ^1^National Engineering Laboratory of Crop Stress Resistance Breeding, School of Agronomy, Anhui Agricultural University, Hefei, China; ^2^Faculty of Science, Universiti Brunei Darussalam, Bandar Seri Begawan, Brunei

**Keywords:** *Triticum aestivum* L, terminal heat stress, grain-filling stage, QTL, genetic regulation

## Abstract

The reproductive compromise under heat stress is a major obstacle to achieve high grain yield and quality in wheat worldwide. Securing reproductive success is the key solution to sustain wheat productivity by understanding the physiological mechanism and molecular basis in conferring heat tolerance and utilizing the candidate gene resources for breeding. In this study, we examined the performance on both carbon supply source (as leaf photosynthetic rate) and carbon sink intake (as grain yields and quality) in wheat under heat stress varying with timing, duration, and intensity, and we further surveyed physiological processes from source to sink and the associated genetic basis in regulating reproductive thermotolerance; in addition, we summarized the quantitative trait loci (QTLs) and genes identified for heat stress tolerance associated with reproductive stages. Discovery of novel genes for thermotolerance is made more efficient *via* the combination of transcriptomics, proteomics, metabolomics, and phenomics. Gene editing of specific genes for novel varieties governing heat tolerance is also discussed.

## Introduction

Wheat (*Triticum aestivum* L.), one of most important cereal crops, is widely cultivated in diverse ecotypes across the world. However, wheat cultivation is often suffering from heat stress damage. According to the IPCC report in 2014, atmospheric temperatures have increased since the beginning of the twenty-first century and are predicted to continue to increase by ~1.0–1.7°C by 2050. As such, wheat growth and development will be subjected to more frequent and severe heat stress as global climate changes (Liu et al., 2017).

Heat stress often occurred during reproduction from flowering to final maturation (Akter and Islam, 2017). It is reported that heat episode during the reproductive phase is fatal to yield and quality of grain by compromising grain setting and grain filling due to lower duration and activities of leaf photosynthesis (Sharkey, 2005), the compromised reproductive development (Farooq et al., 2011), and retarded grain sugar metabolism (Zhang et al., 2018). High temperature results in damages in anther/pollen structure and timing of development (Saini and Aspinall, 1982; Giorno et al., 2013). Pollen development is most sensitive to heat. Pollen dysontogenesis, even abortion, has been reported while exposure to temperatures ≥30°C at this stage (Bheemanahalli et al., 2019; Ullah et al., 2021). Heat stress during rapid grain filling stage can cause photosynthetic capacity reduction, lower metabolic activities, increased oxidative reactive species, and reduced grain filling duration and grain filling rate (Farooq et al., 2011; Akter and Islam, 2017). In addition, high temperature combined with rainfall easily causes pre-harvest sprouting, which is a worldwide problem that reduces wheat yield and quality. The reason is that temperature is one of the most important environmental factors for maintaining dormancy during seed development and for inducing dormancy during seed imbibition (Ali et al., 2019).

Understanding of thermotolerance will help to find solutions to protect heat damage during wheat reproduction, including breeding tolerant cultivars. The physiological mechanisms in controlling the reproductive heat tolerance are focused on the analysis from the activities in both leaf source and grain sink. The enzymes in removing reactive oxygen species (ROS), and heat shock protein (HSP) aggravation, and stay-green traits were shown to be acting to protect heat damage. Meanwhile, efforts have been made to examine genetic markers or genes consistently observed across backgrounds and/or environments with a major or stable effect for heat stress tolerance in wheat. The increasing knowledge of molecular mechanisms of heat tolerance is likely to pave the way for engineering plants with favorable economic yields under heat stress.

## Effects of Heat Stress on Leaf Photosynthetic Capacity and Grain Yield Formation in Wheat

High temperature often occurs at the reproductive stage of wheat, and both carbon source supply and carbon sink intake in wheat are sensitive to heat stress varying with timing, duration, and intensity. Heat stress results in the destruction of photosynthetic systems, which ultimately results in a reduced rate of photosynthesis. Heat stress hindered the formation and development processes of grain, and affected grain filling, grain starch synthesis, eventually resulting into great yield loss.

### Effects of Heat Stress on Leaf Photosynthetic Performance

Photosynthesis is one of most important physiological processes sensitive to elevated temperature (Wahid et al., 2007; Centritto et al., 2011). The major effect on leaf photosynthesis due to heat stress resulted from premature leaf senescence and impaired photosynthetic machinery (Kumar et al., 2010; Vijayalakshmi et al., 2010; Liu et al., 2017).

High-temperature stress may reduce Chlorophyll (Chl) biosynthesis, accelerated degradation, or a combination of both, and therefore, lesser accumulation of Chl from plants. The inhibition of Chl biosynthesis under high temperature is attributed to the destruction of many enzymes (Dutta et al., 2009). For instance, the activity of 5-aminolevulinate dehydratase, an important enzyme in the pyrrole biosynthesis pathway, decreased significantly in wheat under heat stress (Mohanty et al., 2006). High-temperature stress also accelerated degradation of chlorophyll a and chlorophyll b of leaves (Feng et al., 2014; Sattar et al., 2020). Photosynthetic pigments are present in the photosystems, and they are damaged by high-temperature stress, resulting in light absorbing efficiency of both photosystems (PSI and PSII) reduction (Geissler et al., 2009; Zhang et al., 2011). PSII has been considered as an important thermal-sensitive component in photosynthesis than PSI (Feng et al., 2014), which is due to disordering of thylakoid membrane fluidity and dissociation of the light-harvesting complex II from the PSII (Iwai et al., 2010). The inhibition of PSII under heat stress is indicated by a sharp increase of chlorophyll fluorescence (Ristic et al., 2007). The fluorescence induction parameter Fv, Fm, and its ratio are generally used as a response of metabolic disorders under stress. Fv/Fm ratio is an important parameter to determine the maximum quantum efficiency of PSII (Baker and Rosenqvist, 2004; Baker, 2008; Baczek-Kwinta et al., 2011).

Ribulose-1,5-bisphosphate carboxylase/oxygenase (Rubisco) acts as a key enzyme in regulating photosynthesis to heat stress. Rubisco activation (RCA) is a catalytic chaperone involved in modulating the Rubisco activity and heat stress tolerance in wheat (Kumar et al., 2019). In wheat, RCA was inhibited above 30°C; when the exposure of wheat leaf to high temperatures exceeded 40°C, dark or light treatment causes a great change in Rubisco and RCA, and such changes are irreversible under dark conditions (Mathur et al., 2011). RCA protected the nascent proteins from aggregation under heat stress, and removed the inhibitory sugar phosphates from the active site of Rubisco so as to activating it (Portis, 2003). Compared with other enzyme activities, Rubisco is more sensitive to temperature. This is an important reason that high temperature accelerates the rate of formation of dead-end product and decreases the rate of RCA reactivation, finally inhibiting the photosynthesis process (Qu et al., 2017). In addition, the inhibition of photosynthesis at high temperatures is partially attributed to an increase in photorespiration rate (Pinto et al., 2016, 2017). Respiration rate and mitochondrial activities, which are changed by heat stress, show an initial increase with a rise in temperature, reach a critical level, and then decline due to photorespiratory damage (Pinto et al., 2017). Photorespiration of wheat flag leaf significantly increases under heat stress because of changes in solubility of O_2_ and CO_2_ and the affinity of rubisco for these gases (Cossani and Reynolds, 2012).

Consequently, heat stress leads to the synthesis of blocked photosynthetic pigments, membrane disruption, particularly of thylakoid membranes, thereby inhibiting the activities of enzymes and destroying photosynthetic systems (PSII, PSI), which ultimately results in a reduced rate of photosynthesis (Ristic et al., 2008; Rexroth et al., 2011; Ashraf and Harris, 2013).

### Effects of Heat Stress on Reproduction in Wheat

Short-term and prolonged exposure of high-temperature stress compromises grain yield and qualities (Feng et al., 2014). The reduction of grain yield under high temperature is mainly due to the loss of grain number and decreased grain weight (Table 1).

**Table 1:** Effect of high-temperature treatment at different days after anthesis (DAA) on grain number (GN), 1,000-grain weight (TGW) and grain yield (GY) in wheat.

**Variety**	**Method of high temperature**	**Duration**	**Time**	**Temperature**	**GN (%)**	**TGW (g) (%)**	**GY (%)**	**References**
Fleisch 481, Soissons, Plainsman, Magma	Move pots in the phytotron heat stress chamber	10–24 DAA	8 h a day	35°C/20°C	37.3 (6.5↓)	30.6 (25.4↓)	1.2g/spike (30.1↓)	Bányai et al., 2014
		20–34 DAA	8 h a day	35°C/20°C	38.2 (4.3↓)	33.8 (17.6↓)	1.4g/spike (18.1)	
CK	Grown at controlled temperature in greenhouse				39.9	41.0	1.7g/spike	
Yang 16	Free–air temperature enhancement technique in the field	0–34 DAA	0:00–24:00	CK+1.5°C	40.9 (7.3↓)	40.4 (5.6↓)	7258.2 kg·hm^−2^ (13.0↓)	Bian et al., 2012
		0–34 DAA	7:00–19:00	CK+1.5°C	41.6 (5.7↓)	41.2 (3.9↓)	7600.9 kg·hm^−2^ (8.9↓)	
		0–34 DAA	19:00–7:00	CK+1.5°C	43.4 (1.7↓)	41.5 (3.0↓)	7547.1 kg·hm^−2^ (9.5↓)	
		0–34 DAA	0:00–24:00	CK+3°C	38.9 (11.8↓)	39.2 (8.5↓)	6718.2 kg·hm^−2^ (19.5↓)	
		0–34 DAA	7:00–19:00	CK+3°C	40.4 (8.5↓)	39.85 (6.9↓)	7087.2 kg·hm^−2^ (15.0↓)	
		0–34 DAA	19:00–7:00	CK+3°C	39.0 (11.6↓)	40.9 (4.4↓)	6899.8 kg·hm^−2^ (17.3↓)	
CK	Nature condition in the field				44.1	42.8	8342.2 kg·hm^−2^	
Yang 5	Move the pots to a transparent automatic temperature and moisture controlled box	1–3 DAA	08:00–17:00	30°C		34.7 (15.1↓)		Feng et al., 2000
		5–7 DAA	08:00–17:00	30°C		34.1 (16.5↓)		
		12–14 DAA	08:00–17:00	30°C		32.8 (19.7↓)		
		20–22 DAA	08:00–17:00	30°C		31.4 (23.1↓)		
		28–30 DAA	08:00–17:00	30°C		38.2 (6.6↓)		
		20–22 DAA	08:00–17:00	40°C		31.4 (23.1↓)		
		20–22 DAA	08:00–17:00	40°C		31.4 (23.1↓)		
CK1	Nature condition in the field					40.9		
		20–22 DAA	08:00–17:00	30°C		38.5 (8.5↓)		
		20–22 DAA	08:00–17:00	40°C		37.4 (11.0↓)		
CK2	Nature condition in the field					42.0		
Lira–Sa−92, Sakha−8, Gemmeiza−7	Sown late in field					48.1 (15.6↓)	54.9g/plant (24.9↓)	Hassan et al., 2016
CK	Optimal sowing dates in field					57.0	73.1g/plant	
Bainongaikang 58, Luohan 2	Move pots to the walking–in chambers	10–11 DAA	11:00–16:00	38 °C		36.2 (17.7↓)	38.3g/pot (19.1↓)	Jing et al., 2010
		20–21 DAA	11:00–16:00	38 °C		33.2 (24.6↓)	35.9g/pot (24.2↓)	
CK	Nature condition in the field					44.0	47.4g/pot	
Yang 9, Yang 12	Move pots to the artificial intelligence greenhouse	1–3 DAA	8:00–18:00	25°C		37.6 (11.1↓)		Liu et al., 2007
		6–8 DAA	8:00–18:00	25°C		37.4 (11.5↓)		
		13–15 DAA	8:00–18:00	25°C		37.0 (12.4↓)		
		19–21 DAA	8:00–18:00	25°C		36.7 (13.1↓)		
		25–27 DAA	8:00–18:00	25°C		37.3 (11.7↓)		
		33–35 DAA	8:00–18:00	25°C		39.7 (6.0↓)		
		36–38 DAA	8:00–18:00	25°C		40.8 (3.5↓)		
		1–3 DAA	8:00–18:00	30°C		36.2 (14.3↓)		
		6–8 DAA	8:00–18:00	30°C		38.1 (9.9↓)		
		13–15 DAA	8:00–18:00	30°C		37.3 (11.6↓)		
		19–21 DAA	8:00–18:00	30°C		36.2 (14.4↓)		
		25–27 DAA	8:00–18:00	30°C		39.2 (7.3↓)		
		33–35 DAA	8:00–18:00	30°C		41.4 (1.9↓)		
		36–38 DAA	8:00–18:00	30°C		42.4 (0.4↑)		
		1–3 DAA	8:00–18:00	35°C		28.1 (33.5↓)		
		6–8 DAA	8:00–18:00	35°C		27.0 (36.1↓)		
		13–15 DAA	8:00–18:00	35°C		36.3 (14.2↓)		
		19–21 DAA	8:00–18:00	35°C		35.0 (17.2↓)		
		25–27 DAA	8:00–18:00	35°C		35.9 (15.1↓)		
		33–35 DAA	8:00–18:00	35°C		39.5 (6.5↓)		
		36–38 DAA	8:00–18:00	35°C		40.3 (4.5↓)		
		1–3 DAA	8:00–18:00	40°C		19.8 (53.2↓)		
		6–8 DAA	8:00–18:00	40°C		8.8 (79.2↓)		
		13–15 DAA	8:00–18:00	40°C		34.7 (18.0↓)		
		19–21 DAA	8:00–18:00	40°C		33.7 (20.2↓)		
		25–27 DAA	8:00–18:00	40°C		35.0 (17.7↓)		
		33–35 DAA	8:00–18:00	40°C		37.9 (10.3↓)		
		36–38 DAA	8:00–18:00	40°C		39.3 (6.9↓)		
CK	Nature condition in the field					42.3		
Berkut/Krichauff, DH	Late sown in field					25.6 (26.3↓)	1975.8 Kg/hm^2^ (45.8↓)	Tiwari et al., 2012
CK	Normal sown in field					34.7	3647.6 Kg/hm^2^	
Ji 20, Wennong 6	Plastic shed in the field	1–5 DAA	8:00–18:00	CK+3°C	31.2 (21.8↓)	35.2 (2.5↓)	531.8 (22.8↓)	Yang et al., 2014
CK	Nature condition in the field				39.9	36.1	688.5g/m^2^	

#### Grain Setting

Grain setting is sensitive to elevated temperature (Table 1). The temperature favorable for anthesis ranges from 12°C to 22°C in wheat (Farooq et al., 2011). Wheat plants exposed to the abovementioned temperatures can significantly increase floret abortion (Bányai et al., 2014) and reduce the number of spikelet and grains per spike (Semenov, 2009; Kaur and Behl, 2010). Temperature above 30°C during anthesis affects pollen cell and microspore resulting into complete male sterility (Kumar et al., 2014). Even 42°C for 2 h at the anthesis stage could reduce the pollen viability as well as growth of the pollen tip (Kumar et al., 2014). When the duration of heat stress during anthesis is less than 3 days, the anther of wheat florets is structurally abnormal and nonfunctional (Hedhly et al., 2009). In addition, a substantial lowering in grain yield plants was observed when plants are exposed to 30°C for 1–3 days between the beginning of meiosis and anthesis (Kumar et al., 2014). When wheat plants were exposed to high temperature for 20 h, pollen mother cells (PMCs) exposed to 35°C were less likely to progress than those exposed to 30°C, and grain number per spike was reduced at 30°C, and further at 35°C (Draeger and Moore, 2017).

#### Grain Filling, Grain Starch Synthesis, and Grain Quality

Grain-filling duration (GFD) and grain-filling rate (GFR) are important factors in determining the grain yield. High temperature inhibits wheat grain-filling (Jing et al., 2020) and reduces the GFD (Liu et al., 2007). Under high temperature, wheat crop completes its life cycle much quicker than under normal temperature conditions due to accelerated development. Heat stress decreases the grain-filling duration, reducing the time to apoptosis and maturity (Altenbach, 2012). For instance, an increase of 5.4°C above normal temperature reduces the GFD by 8 days in wheat (Tiwari et al., 2012). The time of GFD is shortened to capture resources between anthesis and filling, ultimately reducing the grain yield (Liu et al., 2007; Tiwari et al., 2012). The interesting thing is that high temperature accelerates the GFR, but shortens the GFD; however, under 30°C, the reduced GFD cannot be compensated by high GFR to enhance growth rate (Yang et al., 2014).

Starch makes up ~70% of the dry grain weight; high temperature during grain filling has a great influence on contents and compositions of starch (Wang et al., 2015), and the decrease in yield is mainly attributed to a reduction in the starch content (Li et al., 2017). ADP-glucose pyrophosphorylase (AGPase), soluble starch synthase (SSS), granule-bound starch synthase (GBSS), starch branching enzyme (SBE), and sucrose synthase (SS) are the key enzymes for starch synthesis during grain filling in wheat, and this activity was positively correlated with the starch accumulation rate (Yan et al., 2007; Li et al., 2017). The activity of AGPase, SSS, GBSS, and the content of starch was decreased under high-temperature treatment during grain filling (Yan et al., 2007). Overexpression of the rice soluble starch synthase I (*SSI*) gene in transgenic wheat can improve wheat productivity under terminal heat stress, with the increase in photosynthetic duration and 1,000 grain weight by 21–34% in T2 and T3 transgenic plants compared with the non-transgenic control plants (Tian et al., 2018). Starch accumulation is correlated with the sucrose content of the kernels (Yan et al., 2008); on the one hand, high temperature decreases the inactivation of key enzymes in starch synthesis and inhibits the conversion of sucrose into starch (Asthir et al., 2009); on the other hand, high temperature decreased the time of the maximum grain dry weight and resulted in reduced starch accumulation (Dupont and Altenbach, 2003). High temperature altered the timing of the starch biosynthetic process and resulted in an earlier peak in the gene expression during starch biosynthesis due to an enhanced -amylase activity (Li et al., 2017). A low sucrose content and a decline in the enzymatic activity involved in starch synthesis are responsible for the reduction of starch accumulation (Balla et al., 2011).

Protein is an important characteristic to define grain quality. High-temperature stress affects protein content of the grain, which has a great relationship with leaf nitrogen content (Iqbal et al., 2017). Moderate nighttime warming is more conducive to accumulate protein and increase gluten content in grain, especially the increase of glutenin content (Bian et al., 2012). However, high-temperature stress reduces total protein content, thus shortening the sedimentation time of protein in grains (Labuschagne et al., 2009); meanwhile, high temperature reduces the amino acid level and sedimentation index of grains (Dias et al., 2008).

## The Physiological Mechanisms of Heat Tolerance

Air temperature exceeding certain threshold levels cause excessive accumulation of ROS, oxidative stress, excess membrane damage (Bita and Gerats, 2013; Hasanuzzaman et al., 2013), irreversible degeneration of proteins, and even protein misfolding and raft disruption (Goraya et al., 2017; Lippmann et al., 2019). Plants use various methods to resist heat stress injury, such as mobilizing antioxidant protection system, HSP, phytohormone, prolonging stay-green time, and regulating sugar metabolism of heat tolerance.

### Antioxidant Protection for Heat Tolerance

When the temperature is favorable for the plant, ROS in the form of hydrogen peroxide (H_2_O_2_), superoxide anions (${\text{O}}_{2}^{•-}$), hydroxyl radical (OH•), and singlet oxygen (^1^O_2_) is present in plant vacuoles at lower levels. However, when plants are exposed to temperatures beyond the optimum, ROS level can be significantly enhanced, which will cause negative effects on cell metabolism (Esfandiari et al., 2007). Hence, an efficient antioxidant defense system is important for protecting plants against heat stress (Farooq et al., 2011). Both antioxidant defense system and non-enzymatic antioxidant systems contributed to scavenging ROS. Antioxidant defense system includes superoxide dismutase (SOD), peroxidase (POD), catalase (CAT), ascorbate peroxidase (APX), monodehydroascorbate reductase (MDHAR), glutathione peroxidase (GPX), dehydroascorbate reductase (DHAR), peroxiredoxin (PRX), glutathione S-transferase (GST), and glutathione reductase (GR) (Farooq et al., 2011; You and Chan, 2015); non-enzymic antioxidants include glutathione (GSH), ascorbic acid (AsA), ascorbate, and tocopherols (Farooq et al., 2011; You and Chan, 2015).

### Heat Shock Protein for Heat Tolerance

Heat stress can disturb cellular homeostasis and hindering reproduction in wheat (Sehgal et al., 2018; Hütsch et al., 2019). HSPs play a multifaceted role in plant heat tolerance (Table 2) (Hu et al., 2010; Jacob et al., 2017; Bi et al., 2020), such as protecting proteins from aggregation under heat stress and promoting protein refolding during recovery (Li and Howell, 2021), and involvement in heat stress independent signaling (Liu et al., 2011; Kumar et al., 2016; Jacob et al., 2017; Malik and Lone, 2021). HSPs fall into five categories, namely, HSP110, HSP90, HSP70, HSP60, and small heat shock proteins (sHSPs), respectively (Thomas et al., 2005). It was reported that HSPs regulate the transcription of *HSP* genes (Schoffl et al., 1998); some genes of protective proteins involved in sensing and responding to heat stress during grain filling stage were characterized by overexpression/expression in *Arabidopsis* or wheat (Kumar et al., 2017; Lu et al., 2018). The wheat chloroplastic sHSP (sHSP26) is involved in seed maturation and germination, and greater tolerance to heat stress (Chauhan et al., 2012). Transgenic *Arabidopsis* plants overexpressing *TaHsfA2d* produced greater biomass and grain yield under constant heat stress conditions (Chauhan et al., 2013). Overexpressed *TaHsfC2a*, a transcriptional activator of heat protection genes that serves as a proactive mechanism for heat protection in developing grains, in transgenic wheat, improved the thermotolerance, but did not contribute to dehydration tolerance (Hu et al., 2018). The expression of heat shock factor *Tahsfa6f* is increased in wheat leaves by overexpressing *TaHsfA6f*, which could increase abscisic acid (ABA) levels and enhance tolerance to heat stress in transgenic plants (Bi et al., 2020).

**Table 2:** Heat shock protein genes involved in sensing and response to heat stress.

**Gene**	**Source**	**Trans-host/expression analysis**	**Function**	**References**
*TaHsfA6f*	Cloned the TaHsfA6f gene from the heat and drought tolerant wheat cv. TAM107.	*Arabidopsis*	Through up-regulation of a number of genes involved in ABA metabolism and signaling, and other stress-associated genes.	Bi et al., 2020
*sHSP26*	Cloned the TaHsfA6f gene from bread wheat cv. CPAN1676	Transgenic rice and *Arabidopsis*	Transgenic *Arabidopsis* plants were substantially tolerant under con tinuous high temperature regimen than wild-type plants, as measured by photosystem II (PSII) activity, accumulation of more photosynthetic pigments, higher biomass and seed yield. Transgenic plants produced bold seeds under high temperature, having higher germination potential than the wild-type plants.	Chauhan et al., 2012
*TaHsfA2d*	Homology clone from rice gene rice OsHsfA2d	*Arabidopsis*	A heat shock factor (HSF) gene expressing preferentially in developing seed tissues of wheat grown under high temperatures, possess higher tolerance toward high temperature, also showed higher yield and biomass accumulation under constant heat stress conditions.	Chauhan et al., 2013
*TaHsfC2a-B*	Using the sequences of Hsf DNA-binding domains from rice and *Arabidopsis* Hsf proteins for searching T. aestivum Hsf expressed sequence tags (ESTs) from the NBCI EST database	Transgenic wheat Fielder	A transcriptional activator of heat protection genes and serves as a proactive mechanism for heat protection in developing wheat grains via the ABA-mediated regulatory pathway.	Hu et al., 2018
*HSP90*	Clone from C-306 cultivar of wheat	Expression analysis of HSP90 gene in wheat C-306	A high HSP90 transcript level along with high activities of antioxidant isoenzymes and low proline accumulation is a promising target for developing wheat genotypes with tolerance to heat stress.	Kumar et al., 2013
*HSP70*	Clone from a thermotolerant cultivar C306) of wheat	Expression analysis of HSP90 gene in wheat C-306	The expression of *HSP70* could decrease membrane stability and enhance total antioxidant capacity under heat stress.	Kumar et al., 2016

### Phytohormone for Heat Tolerance

Plant hormones are the endogenous signal molecules that play a key role in the response to the extreme heat during grain filling in wheat (Kumar et al., 2015). ABA is an important signaling molecule under high-temperature stress (Suzuki et al., 2016). ABA reduces the damage of chloroplast structure by preventing photoinhibition and improving PSII efficiency (Li et al., 2020), activating various antioxidant mechanisms by producing various osmolytes, and improving ability of basic and acquired resistance high temperature (Rezaul et al., 2019; Li et al., 2020). For example, ABA induces the expression of NADPH oxidase (RBOHs) in the *Arabidopsis* genome, known as respiratory burst oxidase homologs (RBOHs), to induce ROS (Suzuki et al., 2011; Kaya et al., 2019) and antioxidant protection (Li et al., 2014; Rezaul et al., 2019). In addition, ABA can activate the expression of sucrose transporter gene and metabolism-related genes in heat stress, for instance, sucrose transporter, sucrose synthase gene, and sucrose invertase genes to maintain ATP formation to enhance heat tolerance of plants (Chen et al., 2019; Rezaul et al., 2019). Cytokinins (CTKs) play a key role in the response to temperature stress (O'Brien and Benkova, 2013), which is the most potent general coordinator between the stay-green trait and senescence (Yang et al., 2016), and promotion of grain filling under heat stress (Zavaleta-Mancera et al., 2007; Wang et al., 2012; Hoenig et al., 2018). CTKs protect plants from the deleterious effects of heat stress by activating antioxidant mechanisms, reducing lipid peroxidation protecting photosynthetic apparatus, and delaying senescence (Liu and Huang, 2002). Brassinosteroids (BRs) enhanced activities of enzymes involved in the ascorbate–glutathione (AsA-GSH) cycle and expression of genes encoding these enzymes to resist heat stress; in addition, BRs could increase the production of HSPs against irreversible heat-induced damage (Wu et al., 2014; Jin et al., 2015; Li et al., 2018). Jasmonates (JAs) could activate the defense system to resist heat stress in rice by improved antioxidant enzyme activity, increased proline content, and enhanced osmotic regulation ability (Clarke et al., 2009; Farhangi-Abriz and Ghassemi-Golezani, 2019; Sharma et al., 2019; Yang et al., 2020). The airborne ethylene (ET) may reduce thermotolerance to heat stress by deterring antioxidant defenses (Munne-Bosch et al., 2004). Expression patterns of a heat-responsive gene, *TaGASR1*, revealed that it was strongly induced by stress factors, such as high temperature, drought, high salinity and oxidation, as well as the phytohormones, including methyl jasmonate and ABA, which suggested that the *TaGASR1* gene might participate in these stress and hormone signal transduction pathways.

### Stay-Green Traits Regulating Heat Tolerance

Stay-green, antagonist to senescence, chlorophyll, and photosynthetic capacity of leaves were maintained or prolonged, which is considered an indicator of heat tolerance (Fokar et al., 1998). Since the loss of chlorophyll is associated with senescence, stay-green genotypes are better able to maintain green area of photosynthesis, resulting in a high percentage of carbohydrates. Compared with the stay-green varieties, the yield and inferior grains of no-stay-green cultivar were much more influenced by high-temperature stress (Yang et al., 2014). Moreover, a study showed that high temperatures during reproduction resulted in a significant decline in C and N assimilation and translocation in the heat-susceptible rice (Shi et al., 2013). The stay-green genotype has the characteristics of delayed C-N transfer, or when the transfer occurs, the process of N re-transfer is slower (Thomas and Ougham, 2014). A stay-green cultivar Wennong 6 had relatively higher grain yield under heat stress due to a lower gibberellin (GA_3_) content and a higher zeatin riboside (ZR) content (Yang et al., 2016). The stay-green character showed its potential use in plant breeding, as wheat genotypes in maintaining stay-green of photosynthetic tissues had a greater capacity for grain filling, resulting in increased average weight of grains (Kumar et al., 2010).

### Sugar Metabolism Regulates Heat Tolerance

Previous studies suggested that the high sensitivity of reproductive development to heat stress is attributed to the sugar starvation in non-wheat crops (Frank et al., 2009; Liu et al., 2013, 2019; Ruan, 2014), which could be due to reduced photosynthesis, increased respiration, or compromised sugar unloading into grains (Mittler and Blumwald, 2010; Zhang et al., 2018).

It is reported that soluble sugars have been directly linked to the production rates of ROS (Couee et al., 2006) in regulating ROS metabolic pathways, such as mitochondrial respiration or photosynthesis. Sugars may act as signaling molecules for plant development (Zhang and Zhou, 2013), for instance, higher concentrations of sugars may be ROS scavengers in plants, while lower concentrations of sugars may act as substrates or as a stress signal (Van den Ende and Valluru, 2009). Soluble carbohydrate plays an important role in stabilizing cell membrane and maintaining turgor pressure (Peshev and Ende, 2013). The protective characteristics of soluble sugars during oxidative stress are usually attributed to the production of ROS scavengers and/or repair enzymes triggered by direct or indirect signals (Van den Ende and Valluru, 2009). Fructan and hexose contents in grains were significantly reduced under high-temperature stress (Hütsch et al., 2019), for example, hexose is mainly used in starch synthesis, which directly affects starch accumulation (Hütsch et al., 2019). Further study reveals that sucrose and fructan have premium ROS scavenging properties (Keunen et al., 2013; Peshev and Ende, 2013). Heat stress reduces vacuolar invertase activity in maize grains, thereby preventing sucrose degradation to hexose and reducing starch biosynthesis in the endosperm (Cheikh and Jones, 1995). Due to heat stress leading to oxidative stress, they further proved that galactinol and raffinose at an appropriate concentration have good antioxidant capacity and can protect plant cells from oxidative damage (Nishizawa et al., 2008). However, further study is needed to illustrate how sugar participates in the metabolic process of wheat organisms.

## Breeding Strategies Coping With Heat Stress

To cope with high-temperature stress at the reproductive stage, appropriate measures have been taken to improve crop yield. Strategies to improve heat stress tolerance in wheat include selecting heat tolerance varieties, identifying QTL/genes and exploitation of closely linked markers, and application of closely linked markers in selecting heat-tolerant varieties and marker-assisted breeding in wheat.

### Selection of Heat-Tolerant Varieties

Some traits such as grain yield, 1,000-grain weight, canopy temperature (CT) depression, stay-green, and membrane thermostability that appear to be effective indicators could be used in selecting heat-tolerant varieties, though there is no direct screening method to select heat-tolerant varieties (Ni et al., 2018). Stable yield performance of genotypes under heat stress conditions is vital to identify heat-tolerant genotypes (Mason et al., 2010). Thus, the relative performance of yield traits under heat-stressed and non-stressed environments has been widely used as an indicator to identify heat-tolerant wheat genotypes (Sharma et al., 2016). The heat susceptibility index (HSI) was shown to be a reliable indicator of yield stability and a proxy for heat tolerance (Geng et al., 2016; Chen et al., 2017; Zhang et al., 2020). Twenty-six wheat varieties with stable heat resistance were screened using the HSI of 1,000-grain weight and yield all less than 1; even 11 varieties had relatively strong heat resistance with HSI less than 1 in consecutive years (Zhang et al., 2020). Using integrated HSI of yield-related traits and cell membrane thermostability as selection criteria, seven wheat varieties (lines) were selected with heat tolerance (Geng et al., 2016). Leaf senescence is an early response to heat stress, so delayed senescence/stay-green genotypes are important germplasm for resistance heat stress (Abdelrahman et al., 2017). Compared with non-stay-green varieties, stay-green varieties are of agronomic interest, as although the photosynthetic capacity is maintained, the onset of senescence is delayed or the development of senescence is slowed under heat stress (Thomas and Ougham, 2014). CT can be successfully used as an important selection parameter in breeding program at field, which showed a significant and negative correlation with grain yield, biomass, and 1,000-grain weight, and a positive correlation with spike number per plant during wheat growing period, especially after flowering (Gautam et al., 2015). Lower CT during late grain-filling protected chlorophyll and photosynthesis, exhibiting a greater degree of tolerance to terminal heat (Gautam et al., 2015).

Based on these conventional methods, a series of heat-resistant varieties were selected from existing varieties (Table 3), and these germplasm resources were defined as carriers with actual or potential utilization value and biological genetic information. However, traditional phenotypic screening for heat stress tolerance in wheat is slow, laborious, and expensive, which has trailed the high-speed development of genomics and transcriptomics, thereby restricting crop breeding and functional genomics study. More attention needs to be paid to high-precision and high-throughput phenomics studies, in particular, phenomics and multiomics joint analysis in identifying heat-tolerant germplasm resources (Cobb et al., 2013; Zhou et al., 2018). High-throughput phenotypic screening is faster and can capture more genetic information and comprehensive information for the effect of injuries caused by heat stress (Crain et al., 2018; Schmidt et al., 2020). Crain et al. (2018) evaluated a portable phenotypic system named “Phenocart,” which was used to record more than 1.1 million phenotypic observations in 1,170 wheat germplasm resources under drought and heat stress, and more than 2,000 GBS markers were identified and genotyped using genotyping sequencing (GBS) technique (Crain et al., 2018). An X-ray computed tomographic analysis was carried out on 203 diverse wheat accessions under heat stress. It takes only 7 min per ear to scan the main shape of the seed (smaller, shriveled seeds with an increased seed surface), and computed tomography evaluating grain set with an accuracy of 95–99% (Schmidt et al., 2020). The application of high-throughput plant phenomics, especially for abiotic stress, will greatly accelerate breeding efficiency. They suggest that advances in yield prediction models and the ability to generate data from genomic and phenotypic data will make these selection strategies easy to adopt by plant breeders for improved genetic gain rates.

**Table 3:** Heat-tolerant and heat-sensitive wheat genotypes and the selected indicators and performance to high-temperature stress.

**Selected Indicators**	**Heat stress method**	**Heat stress time**	**Heat tolerant genotypes**	**Performance of tolerant genotypes**	**Sensitive genotypes**	**Performance of sensitive genotypes**	**References**
Heat susceptibility index estimated for 1000 grain weight, grain yield per plant, grain weight of the main-spike and flag leaf senescence scale	Artificial temperature-rising facility made from hollow steel pipes covered with a white polythene plastic film	DAA7-21, 9.00–17.00	Gaoyou 9415, Hemai 13, Hindi62, Jimai 22, Kexin 9, Shannong 8355, Taishan 23, Yannong 5286, Zimai 7,	The HSI values were less than 1 for thousand kernel weight, grain yield per plant and grain weight per spike			Cao et al., 2015
Canopy temperature, grain yield and its components	Late and very late sown conditions.	Terminal heat stress	HI 8627, HI 8638, HI 8498, HI 896, HI 8691, MACS 3125	Lower canopy temperature, Higher grain yield/plant, biomass/plant, harvest index and test grain weight over years			Gautam et al., 2015
Heat susceptibility index, geometric mean yield and cell membrance thermostability	Under plastic film covered shelter	Began at the 15th day after flowering to harvest DAA15-mature	Nongda 212, Heng 6632, Nongda 3492, Jimai 19, Nongda 413, Nongda 2149, Hengguan 216	Lower heat susceptibility index, higher geometric mean yield and better cell membrance thermostability			Geng et al., 2016
HSI of thousand-kernel weight	Under plastic film covered shelter	From DAA10 to mature	Nongda 189, CA0518, and Jingdong 8	Performed high yield and high 1000 grain weight under both normal and heat-stress environments	Nongda 211, Shimai 15, Jimai 22, Nongda 3432, and Shannong 2149	Performed high yield and high TKW in normal environments, but low yield and low TKW in heat stress environments, and were characterized with poor resistance to heat stress.	Han et al., 2010
Chlorophyll content, grain quality and adversity index of thousand-kernel weight	The artificial intelligence greenhouse	DAA10-20, 9:00–16:00	Shannong 23, Zhoumai 18, Taishan 9818		Shiluan 02-1, Jinan 17	The chlorophyll content in flag leaf of wheat cultivars and thousand-kernel weight decreased, protein content was significantly increased while the starch content was significantly decreased.	Li et al., 2017
Membrane stability index, SPAD value, Fv/Fm ratio and Pn	Sown late	From flowering to grain maturity	DBW 14, RAJ 3765, HD 2643 and HALNA	Higher membrane stability index, chlorophyll content, photosynthesis rate, harvest index under heat stress conditions	HD 2987, SHANGHAI, HD 2402 and WH 730	Lower membrane stability index, chlorophyll content, photosynthesis rate, harvest index under heat stress conditions	Nagar et al., 2015
Stay-green character, water use efficiency, grain filling rate, grain filling duration, grain yield and harvest index	Placing pots in glass canopies with temperature of 4–5°C above than the ambient until maturity	The heat stress was imposed separately at booting, heading, anthesis and post anthesis stages until maturity.	Mairaj-2008	Stay green and take more duration for grain filling	BARS-2009, Shafaq-2006	Poor grain filling and less grain yield, rapid leaf senescence	Nawaz et al., 2013
The adversity resistance indices of functional period of flag leaf and 1,000-kernel weight	In the greenhouse	DAA10-20;09:00–16:00	Shannong 23, Liangxing 77, Shannong19, Luohan 7, Chang 4738	Both normalized greenness intensity of wheat canopy and 1,000-kernel weight were decreased, functional period of flag leaf was shorted, the grain protein content significantly increased and starch content significantly reduced	Jinan 17, Jimai 20, Shiluan 02-1, Zhoumai 24		Yi et al., 2015
Heat susceptibility index of 1,000 grain weight	Plastic film covered shelter	From flowering to grain maturity	Xinchun 37, Xinchun 2, Xinchun 38	HSI less than 1	Xinchun 13, Xinchun 18, Xinchun 33	HSI more than 1	Zhang et al., 2020

### Identification of QTL/Genes Related to Heat Tolerance

Over the last three decades, efforts have been made to elucidate the genetic basis of heat stress during grain filling (Ni et al., 2018). The QTLs associated with heat tolerance were identified on all 21 wheat chromosomes in wheat (Table 4; Pinto et al., 2016; Ogbonnaya et al., 2017; Bhusal et al., 2018), of which, some of the QTLs detected by different researchers or under different genetic backgrounds were located at the same or similar regions. QTLs for stay-green related traits were identified mainly on chromosome 2A and 7D, and several important QTLs mainly associated with yield and CT were detected on chromosome 3B. This indicates that certain chromosomal regions may have genes closely related to heat tolerance in wheat, and these promising molecular markers could be used in wheat molecular assisted breeding in the future. For example, 14 SSR markers linked to some important traits, including grain filling duration, HSI grain filling duration, HSI single kernel weight of main spike, and HSI kernel weight under heat stress have been used to screen varieties for heat tolerance (Sadat et al., 2013). Recently published sequencing information will be of great benefit for map-based cloning of major QTLs controlling heat tolerance during grain filling (IWGSC RefSeq 1.0; https://urgi.versailles.inra.fr/blast_iwgsc/blast.php; IWGSC, 2018). Most of these QTLs were identified during grain-filling stage, while fewer QTLs were identified for heat stress tolerance during flowering in wheat. Only a genome-wide association analysis of spike ethylene under heat stress at the anthesis stage was reported using an Illumina iSelect 90K SNP genotyping array in 130 diverse wheat elite lines (Valluru et al., 2017).

**Table 4:** QTLs for traits associated with terminal heat tolerance in wheat.

**Chromosome**	**Markers**	**Similar QTL region**	**Stable or major QTL**	**Phenotypic variation explanation (PVE)**	**Associated trait**	**References**
1B	*gwm11, gwm268*	*		10.6–11.0%	Grain filling duration, kernel yield	Yang et al., 2002; Mason et al., 2010; Sharma et al., 2016
	*wPt3477, Xbarc119*	*		10.0%	Yield, green leaf duration	Naruoka et al., 2012; Pinto et al., 2016
	*wmc419, wmc156*	*		9.0–10.0%	Flag leaf wax content, spike non-glaucousness	Dubcovsky et al., 1997; Mondal et al., 2015
2A	*gwm356, XCGT.TGCG-349*		*	10.0–26.0%	Senescence-related traits	Vijayalakshmi et al., 2010
	*nine QTLs (close to gwm372)*		*	3.81–18.05%	Chlorophyll fluorescence and chlorophyll content	Bhusal et al., 2018
2D	*gwm261, gwm484*	*		More than 11.0%	Temperature depression of main spike, flag leaf length and width,	Tiwari et al., 2013
	*wPt-0153, wPt-730427*	*		5.5–5.61%	Yield-related traits	Edae et al., 2014; Ogbonnaya et al., 2017
3B	*wmc527, wmc326*		*	19.0–21.2%	HSI of yield components	Mason et al., 2010
	*wPt-9388, wPt-8021*	*	*	Up to 22.0%	CCanopy temperature, grain yield	Pinto et al., 2010; Bennett et al., 2012
	*barc229, barc164*	*	*	4.5–9.0%	Temperature depression of main spike, HSI_single kernel weight main spike	Mason et al., 2011; Mondal et al., 2015
	*wPt-1940, barc0164*	*	*	7.0–13.5%	Total green biomass, the velocity of greenness loss and the proportion of plant greenness lost mid grain filling, green leaf duration	Naruoka et al., 2012; Pinto et al., 2016
5A	*Xgwm293, gwm639, Vrn-A1*	*		12.0%	Grain filling duration; early and late grain filling efficiency	Yang et al., 2002; Acuna-Galindo et al., 2015; Sharma et al., 2016; Ogbonnaya et al., 2017
6D	*cfd42*	*	*	32.1%	Spike temperature depression	Mason et al., 2010, 2011
7B	*Xgwm1025–Xgwm745, Xgwm577*	*	*	10.4–20.3%, 25.0%	Canopy temperature depression	Barakat et al., 2011; Paliwal et al., 2012
7D	*acc/cat-10*	*		15.0%	Stay-green, grain filling, canopy temperature and days to heading, permanence of greenness	Kumar et al., 2010; Vijayalakshmi et al., 2010; Pinto et al., 2016

The meaning of * is “Similar QTL region” and “Stable or major QTL”.

#### QTL Clusters in Same or Similar Regions

Most of the QTLs were found to be associated with heat tolerance in wheat grain filling, and there existed QTL clusters in same or similar regions on chromosome 1B, 2D, and 5AS (Table 4). Same QTL region on 1B was identified with heat tolerance for grain-filling duration (Yang et al., 2002; Mason et al., 2010; Acuna-Galindo et al., 2015; Sharma et al., 2016) and for kernel weight (Mason et al., 2010) closely linked to markers *gwm11* and *gwm268*. A strong QTL for yield found on 1B (Pinto et al., 2016) co-located with a QTL for green leaf duration flanking markers *wPt3477* to *Xbarc119* was detected in spring wheat grown under heat stress in greenhouse experiments (Naruoka et al., 2012). A stable QTL for flag leaf wax content was identified on chromosome 1B flanked with *wmc419* and *wmc156* (Mondal et al., 2015), which was co-located with a QTL for non-glaucousness spike in a similar position (Dubcovsky et al., 1997). Two HSI QTLs mapped to chromosome 2D were linked with *gwm261* and *gwm484* located closely with a GFD QTLs on chromosomes 2D (Tiwari et al., 2013). The yield-related marker-trait associations (MTAs) identified on chromosome 2D between 96 and 104 cM (Ogbonnaya et al., 2017) were previously identified as stable MTAs for grain yield using 9 K SNP markers within the same region (Edae et al., 2014). *Hgfd.iiwbr-5A* and *QLgfd.iiwbr-5A* associated with early and late grain filling efficiency detected by Sharma et al. (2016) were also located on the short arm of chromosome 5A, which is very close to a meta-QTL MQTL39 (close to *gwm639*) for grain filling reported by Acuna-Galindo et al. (2015).

#### Important QTLs Mainly Associated With Yield and Canopy Temperature

Numerous important QTLs controlling heat tolerance during grain-filling were identified on chromosome 3B, and were mainly associated with yield and CT (Table 4). An important QTL region flanked with markers *wmc527* and *wmc326* on chromosome 3B was identified to be associated with HSI of yield components explaining 19.0–21.2% genetic variance using a Halberd × Cutter RIL population (Mason et al., 2010). Two key QTLs were detected on chromosome 3B using a set of 255 doubled haploid lines, which had a large effect on CT and grain yield, accounting for up to 22% of the variance for these traits; in particular, the locus on chromosome arm 3BL had its largest effect under the heat stress conditions, with the RAC875 allele increasing grain yield by 131 kg/ha (Bennett et al., 2012), while Pinto et al. (2010) also detected a QTL of relatively large effect in a similar region under similar heat stress conditions. One stable QTL linked with markers *barc229* and *barc164* influencing HSI single kernel weight main spike and temperature depression of main spike was mapped on chromosome 3B using the same Halberd × Karl92 RIL population across environments (Mason et al., 2011; Mondal et al., 2015). In agreement with the results of Pinto et al. (2016), in the Seri × Babax RIL population on 3B chromosomes seemed to contain genes driving total green biomass, the velocity of greenness loss, and the proportion of plant greenness lost in the middle of grain filling. Naruoka et al. (2012) also found that the 3B chromosomes controlled duration-related QTL of green leaf in spring wheat grown under heat and drought stress. In addition, major and stable QTLs contributing 10.4% ~ 32.1% phenotypic variation for spike temperature depression and CT depression were detected on chromosome 6D in the Halberd × Karl92 population RILs (Mason et al., 2010, 2011) and on chromosomes 7B in the NW1014 × HUW468 RILs (Paliwal et al., 2012) (Table 4).

#### QTLs for Stay-Green Related Traits

Stay-green is reported to be induced by heat stress and the duration of the leaves staying green is dependent on the genetic background (Kumar et al., 2010). QTLs for stay-green related traits under heat stress were identified mainly on chromosome 2A and 7D in wheat (Table 4). QTLs for such traits were mapped on chromosome 2A within markers interval *gwm356* and *XCGT.TGCG-349* (Vijayalakshmi et al., 2010). Nine QTLs were clustered on chromosome 2A affecting chlorophyll fluorescence and chlorophyll content; *Qchc.iiwbr-2A*, linked with marker *gwm372* that explained 3.8–18.1% of phenotypic variation, was the consistent QTL on the same locus (Bhusal et al., 2018). The maximum phenotypic variance of 15.0% QTL linked with marker *acc/cat-10* was detected on chromosome 7D associated with stay-green, which was co-located with the QTL for grain filling, CT, and days to heading (Pinto et al., 2016), and this locus has been previously described as associated with permanence of greenness under high temperatures in wheat (Kumar et al., 2010; Vijayalakshmi et al., 2010).

#### Putative Genes Were Revealed in Response to Heat Stress in Wheat

Discovery of novel genes is made more efficient *via* the combination of transcriptomics, proteomics, metabolomics, and phenomics; some putative genes were revealed by omics techniques in response to high-temperature stress in wheat at the grain filling stage (Table 5). Differentially expressed proteins through proteomics approach or transcript profiling are mainly involved in carbohydrate metabolism (Majoul et al., 2003; Laino et al., 2010), starch synthesis (Majoul et al., 2003), ATP synthesis (Majoul et al., 2003; Wang et al., 2015), HSPs (Laino et al., 2010; Wang et al., 2015), photosynthesis (Wang et al., 2015; Kumar et al., 2019), and some defense-related proteins (Laino et al., 2010), translation initiation factors (Majoul et al., 2003), and antioxidant enzymes (Wang et al., 2015). Whole transcriptome analysis of thermotolerant wheat *HD2985* found a putative Rubisco to be significantly upregulated under terminal heat stress. A positive correlation was established between the RCA enzyme activity and radical scavenging potential in the leaves of wheat (Kumar et al., 2017, 2019). Similarly, a novel candidate gene *miR430* on 3B was found using *de novo* assembly and cloned from wheat cv. *HD2985*, which can be used to manipulate the expression of target genes under heat stress toward enhancing thermotolerance for the development of “climate-smart” wheat crop (Kumar et al., 2017). *TaBI-1.1*, a wheat *BI-1* conserved gene, and *TaFKBP62*, a *TaBI-1.1*-interacting protein that colocalized with *TaBI-1.1* on the endoplasmic reticulum membrane and enhanced heat stress tolerance, were identified by an RNA sequencing analysis of heat-treated wheat *Xiaobaimai* (Lu et al., 2018). In addition, *TaZnF* which belongs to C4HC3-type zinc finger transcription factor was found to be highest in the seed and it starts at the post anthesis period 3–5 DAA. Overexpression of *TaZnF* in *Arabidopsis* thaliana conferred improved tolerance to heat during their growth and development, had larger primary roots, more lateral branching, bigger, and more numerous leaves, resulting in more yield (Agarwal and Khurana, 2018). *TaWRKY1* and *TaWRKY33* transgenic wheat plants exhibited enhanced tolerance to heat stress (He et al., 2016).

**Table 5:** Putative genes for heat stress in wheat.

**Gene**	**Source**	**Expression analysis**	**Function**	**References**
*TaZnF*	Full CDS of *TaZnF* was cloned in pGBKT7 vector	*Arabidopsis*	Play important roles in various plant processes including regulation of growth and development, signaling networks, responses to abiotic stresses etc.	Agarwal and Khurana, 2018
*TaWRKY1*; *TaWRKY33*	Clone from wheat Xiaobaimai	Wheat	*TaWRKY1* was slightly up-regulated by high-temperature and abscisic acid (ABA), and down-regulated by low-temperature. *TaWRKY33* was involved in high responses to high-temperature, low-temperature, ABA and jasmonic acid methylester (MeJA).	He et al., 2016
*miR430*	miRNome analysis from wheat HD2985	Wheat	Negative regulation of the target gene expression in response to terminal heat stress.	Kumar et al., 2017
*RuBisCo activase (Rca) gene*	Whole transcriptome analysis	Wheat	A positive correlation was established between the Rca enzyme activity and radical scavenging potential in the leaves	Kumar et al., 2019
*TaBI-1.1*	RNA sequencing analysis from wheat Xiaobaimai	*Arabidopsis*	Co-localized with *TaFKBP62* on the endoplasmic reticulum (ER) membrane and enhanced heat stress tolerance.	Lu et al., 2018

Although these genes have been shown to be involved in heat stress signaling, more components remain to be identified and characterized in effectively elucidating the mechanism of thermotolerance. Gene editing techniques have been used to improve abiotic stress resistance of crops. By knocking out *OsARM1, OsNramp5*, and *OsHAK1*, breeders have developed rice strains with low levels of cadmium, radioactive cesium, and arsenic, respectively (Nieves-Cordones et al., 2017; Tang et al., 2017; Wang et al., 2017). In 2018, research on the OsPYL abscisic acid receptor gene family revealed that pyl1/4/6 triple knockout rice created by CRISPR/Cas9 editing had increased grain yield, greater high-temperature tolerance, and reduced pre-harvest sprouting compared with the wild type (Miao et al., 2018). This provides a possibility for the application of gene editing technology in heat tolerance enhancement and molecular level mechanism analysis of wheat, which requires attention in the future.

## Conclusion and Future Perspectives

The high temperature during wheat reproductive stage has been receiving increased attention due to climate change. This review assessed the effects of heat stress on leaf photosynthetic capacity and grain yield formation in wheat, the associated physiological mechanisms of heat tolerance, and the breeding strategies. Heat stress during the reproductive stage causes great loss to wheat production through compromise in both photosynthetic capacity and sink size and activities. The heat during anthesis is fatal for grain setting through disturbing reproductive success, while the heat after anthesis reduces starch content by decreasing the activity of key enzymes in starch synthesis and grain filling duration. Antioxidant system, HSPs, and hormones were stimulated to protect the damage to cell and enzyme integrity from heat stress. The trait of staying-green acts together in protecting heat damage.

Identified genomic region and genes will play an important role for wheat improvement in terms of introgression of heat-tolerant genes/QTLs into an elite variety or pyramiding of all heat-tolerant genes into an agronomically superior variety/genotype, which will provide markers to assist selection for breeding strategy. It is noted that most of QTLs or genes were identified mainly associated with grain filling under heat stress. Hence, more research about the QTLs detected with traits of seed setting under high temperature should be done in the future. In addition, candidate genes involved in regulating heat tolerance will be available in breeding heat-tolerant varieties using the gene editing technology. The great potential of plant phenotyping in identification of more valuable traits using high-throughput image system facilitates to clarify the molecular network of heat tolerance. As such, this overview provides a thorough understanding of the impact of heat stress from leaf source to grain sink, protection mechanisms from heat damage, as well as associated molecular regulation and breeding contribution.

## Author Contributions

YZ and YS conceived this review. ML, JF, and YZ collected information and drafted this review. ML, JF, and HZ drafted tables. ML, UN, JL, and YS finalized the study. All authors read the manuscript and approved it for publication.

## Funding

The study was supported by grants from the National Natural Science Foundation of China (No. 31901540), the Anhui's University Natural Science Research Project (No. KJ2019A0175), and the National Key Research and Development Plan Program of China (No. 2017YFD0300204-3).

## Conflict of Interest

The authors declare that the research was conducted in the absence of any commercial or financial relationships that could be construed as a potential conflict of interest.

## Publisher's Note

All claims expressed in this article are solely those of the authors and do not necessarily represent those of their affiliated organizations, or those of the publisher, the editors and the reviewers. Any product that may be evaluated in this article, or claim that may be made by its manufacturer, is not guaranteed or endorsed by the publisher.
